# Thermal stress information as a tourism-oriented climate product: Performance analysis for selected urban destinations in Romania and Italy

**DOI:** 10.1016/j.heliyon.2024.e24682

**Published:** 2024-01-13

**Authors:** Liliana Velea, Zenaida Chițu, Roxana Bojariu

**Affiliations:** a National Meteorological Administration, 013686, Bucharest, Romania; b Dept. of Humanities, Ca’Foscari University of Venice, 30123, Italy

**Keywords:** Thermal comfort, Reanalysis, Heat stress, Climate service, Tourism

## Abstract

The study addresses the characteristics of a climate service targeting tourists and discusses the evaluation of its products with a particular focus on the thermal stress information. Furthermore, an assessment of the impact of input data on the accuracy and relevance of the thermal stress product is presented. The thermal stress is expressed through UTCI (Universal Thermal Climate Index) and it is computed from UERRA regional reanalysis and E-OBS gridded dataset, for summer season during 2011–2018. The analysis targets 10 cities with different characteristics located in Romania and Italy. It focuses on the impact of three temperature-related input data (instantaneous temperature at 12:00 UTC, daily maximum and daily mean temperature) on the thermal stress intensity. The results show that differences up to 4 days in the pronounced thermal stress category may appear when employing daily maximum temperature compared to the use 12:00 UTC instantaneous temperature, while the use of daily mean temperature leads to strong underestimation of thermal stress in this category. The findings are of interest in defining the technical choices of products to be incorporated in a climate service for tourism in order to assure a good user uptake.

## 1 Introduction

Thermal stress is an important attribute of weather with high impact on people's health. Heat stress has been shown to be correlated with increased incidence of heat strokes, coronary heart diseases and even deaths due to preexisting cardio-vascular and cerebrovascular diseases [[1], [2], [3], [4]]. Cold stress is also associated with health issues [[5], [6], [7]].

Due to its practical importance, information on thermal stress is disseminated toward population on continuous basis by most of national meteorological centers. Thermal stress is also part of the forecasted products available through various meteorological apps (e.g., Refs. [[8], [9], [10], [11]]). The forecasted information on thermal stress is useful for population and implicitly for tourists, for the latter pertaining to the planned or on-going activities during the vacation. However, climatic information on thermal stress, of interest for planning ahead the vacation, is not easily available for tourists, despite their interest for information at this time scale [12]. In some degree, this is valid for other types of climate information as well, which might be of interest and of help for tourists if customized to answer their needs.

Solution to this issue is provided by climate services, seen as ‘a decision aide derived from climate information that assists individuals and organizations in society to make improved ex-ante decision-making’ [13] or, in a larger sense, as ‘the provision of climate information for use in decision-making’ [14]. European initiatives like ERA4CS [15], PLACARD [16], Copernicus Climate Change Services (C3S) [17] fostered the design of the research framework for the development of a large palette of climate products and services. Several services, offered by public or private entities, are available, targeting economic sectors like agriculture [18], energy [19], water management [20], risk management [21], urban climate resilience and adaptation [22,23].

Climate services targeting tourism sector are relatively limited in number and their focus, just like in other socio-economic sectors, is more toward public decision-making actors [24] addressing especially issues related to climate forecasting (e.g., at seasonal scale) and climate change (i.e., for adaptation purposes) [25,26]. But a climate service may target as well another ‘market’ segment than the authorities or stakeholders, namely individual users. In this case, the climate information used as basis for the products included in the service would focus more on historical data and at most on seasonal forecasting, as at the individual level the climate-related decisions to be made have impact on shorter periods of time than those associated with climate change (e.g., in tourism – planning vacations; in agriculture – planning crops and associated activities for the next season or year). Examples of such services oriented toward tourists include the climate service for surf described by Boqué Ciurana and Aguilar (2021) [27] or the 'ClimApp' tool [11] providing personalized thermal stress information which may also be relevant for tourism purposes, although not for ahead-planning process. In this line, WeCENT project [28] developed a prototype of climate service targeting individual tourists, with the potential to be extended also to other end-user types (e.g., tourism investors, managers). The prototype includes 12 tourism-customized information, adapted for 40 urban, rural, mountain and beach destinations in Italy and Romania. The climate products provided through the prototype service are built using climate reanalysis, satellite-based products, analysis and forecast information. The selection of the information type is based on literature findings and it has been updated taking into account the tourists preferences identified through a survey [12]. The latter showed that thermal stress is the climate feature of most interest for the respondents independent of the destination type and consequently it has been introduced in the prototype service, along with other 3 biometeorological climate information.

The availability of climate services does not guarantee their user uptake, which in turn may be seen as a first evaluation measure of the service quality and ‘success’. Several studies (e.g., Refs. [29,30]) highlight the barriers to overcome the limited inclusion of climate information in the decision-making process. In the tourism sector, EU-MACS project [31] identified ‘constraints and enablers shaping climate services take-up in the Austrian and Finnish tourism sector’, among these being the need to demonstrate and communicate the benefits of using climate services. In this line, the efforts to define an evaluation framework (e.g., Refs. [[32], [33], [34]]) and to evaluate climate services with regard to their uptake [35], technical aspects [36] or economic/societal benefits (e.g., Refs. [[37], [38]]) have been intensified. These studies apply to climate services already implemented and for which data needed in the evaluation was available.

Discussions on the evaluation of individual climate products included in a climate service are dependent on the definition of ‘climate product’. The most frequently encountered definition is related to the data – a quality-checked dataset, independent of the source of the data (e.g., measurements, satellite-based observations, models) is usually considered as a climate product. In this context, the evaluation of individual ‘climate products’ is well represented, as almost any dataset made available for the research community is soundly examined and documented with regard to its scientific robustness (e.g., methodology, accuracy, uncertainty etc.). In other cases, the climate product may be a complex ensemble of information for example ‘covering the nexus of climate, agriculture, and food security’ [39] or provided through an ‘online decision support tool’ [40].

For the particular case of climate services targeting individual tourists there is no publicly available information useful for their evaluation, for the moment. Nevertheless, an evaluation of the performances of the products included in the service may bring useful insights for identifying their strengths and weaknesses which may be further reflected in the service characteristics, for establishing potential directions of products/service improvements and possibly for identifying new market segments for the developed information.

The present study starts from this gap regarding the evaluation of climate products targeting tourists and its aim is twofold: firstly, to identify possible approaches for a performance analysis of a climate product included in a tourism-oriented (climate) service, with a particular focus on the thermal stress information; secondly, to investigate the performance of the product based on thermal stress in one of the previously identified instances. In order to achieve this, the thermal stress product included in the WeCENT prototype climate service is used as reference. The performance analysis explores technical aspects of the product expressed through UTCI (Universal Thermal Climate Index) [41] and it focuses on the impact of input temperature-related information on the characterization of thermal stress intensity. The analysis makes use of one temperature-related information available from UERRA reanalysis dataset and two available from E-OBS observation-based dataset, being applied to the 10 urban destinations included in the WeCENT prototype service. The performance analysis fits the objective of examining the impact of the input data with regard to the user needs, also proposing possible solutions to mitigate the limitations in the input data. The findings may be relevant for the development process of climate products to be incorporated in a service, especially for its early stages and for the design of the marketing strategy of such products and services.

## 2 Thermal stress expressed through UTCI

The most widely used definition of thermal comfort is given by the American Society of Heating, Refrigerating and Air-Conditioning Engineers and it states that thermal comfort is “the state of mind, which expresses satisfaction with the thermal environment” [42,43] thus being a subjective perception of the external thermal conditions. Numerous attempts to express thermal comfort as a function of weather/climate conditions are recorded in literature, such that about 165 indices have been defined [44].

Universal Thermal Climate Index is one of the 4 indices used most frequently in research [44,45], being employed for example in studies related to health (e.g., Refs. [46,47]), tourism (e.g., Refs. [48,49]), climate and climate change studies [[50], [51], [52], [53]]. It is based on human heat balance models and it is designed to be applicable in all seasons and climates and for all spatial and temporal scales [41] as long as the domain of definition for input parameters values is preserved [54].

UTCI is an ‘equivalent temperature’ [55] derived with the use of several meteorological parameters – air temperature at 2 m (T2m), relative humidity (RH), wind speed at 10 m (w10 m) and radiation (mean radiant temperature at 2 m, denoted in the following as Tmrt) and it is associated with an assessment scale including 10 comfort categories (Table 1, columns 1 and 2). The index may be computed using a Look Up Table (LUT) approach or a 6th order polynomial regression equation; the first method is more accurate, while the second is computationally faster and with ‘acceptable accuracy for most conditions, with the approximation error increasing for high wind speeds above 17 m/s’ [54]. Previous studies (e.g. Refs. [41,56]) show that the relative humidity has a minor impact on thermal stress (UTCI) except in humid environments, while the wind is more important in association with low temperature.

**Table 1:** Thermal comfort classes associated with UTCI values.

UTCI (^o^C) range	Stress category	Categories used in the final product
>46	Extreme heat stress	Pronounced hot stress
38 ÷ 46	Very strong heat stress	
32 ÷ 38	Strong heat stress	
26 ÷ 32	Moderate heat stress	Acceptable thermal stress
9 ÷ 26	No thermal stress	
0 ÷ 9	Slight cold stress	
−13 ÷ 0	Moderate cold stress	
−27 ÷ −13	Strong cold stress	Pronounced cold stress
−40 ÷ −27	Very strong cold stress	
< −40	Extreme cold stress	

## 3 Potential approaches to performance analysis of a tourism-oriented climate product -case study: thermal stress

Before discussing its characteristics, the expression ‘tourism-oriented climate product’ should be defined. In marketing theory, a product is ‘anything that can be offered to a market for attention, acquisition, use, or consumption that might satisfy a need’ [57]. A more tourism-related definition, as given by United Nations World Tourism Organization (UNWTO) [58], states that ‘Tourism Product is a combination of tangible and intangible elements, such as natural, cultural and man-made resources, attractions, facilities, services and activities around a specific center of interest which represents the core of the destination marketing mix and creates an overall visitor experience including emotional aspects for the potential customers. A tourism product is priced and sold through distribution channels and it has a life-cycle.’

Starting from these definitions, in the context of the present study the expression ‘tourism-oriented climate product’ is used for describing a scientifically-based climate-related information targeting tourists interests and customized to answer their needs, which may or may not be priced.

Ideally, a tourism-oriented climate product would be the result of co-creation, which implies the utilization of input from users (tourists) to design it (e.g., Ref. [59]); alternatively, it may be developed iteratively - it may start from the existing literature on tourists preferences for climate features, proposing its basic elements, assess the product against tourists' opinions and restart the process based on the latter, in order to update it and get it closer to the tourists’ needs.

Any which way, in order to insure a good user uptake, the products included in the climate service have to answer to user needs. This requirement expands further on several directions: content (e.g., to include information of interest for the users), accuracy (e.g., high scientific quality; information relevant at the temporal and spatial scales of interest for the users), presentation form (i.e., the information to be disseminated in an easy-to-use form), delivery method (e.g., single access point for all relevant information, mobile/desktop apps), price. The spatial scale should be appropriate to describe the specific touristic destination (i.e., at destination level) [60], thus the information in the climate product should be developed ideally from long-term local measurements of relevant weather parameters for each destination of interest. The relevant temporal scale depends on the targeted segment of vacation planning process: for ahead planning (e.g., where should we go next year?) climate scale (i.e., averages over several years) would be relevant; for near-time planning (e.g., what should we do tomorrow?) short-term forecast would be more appropriate. Regarding the climate scale, here also may be important to acknowledge the difference between the standard climate period used in research (i.e., 30 years) and the subjective perception of people on what ‘climate’ means, especially in the context of touristic activities. Studies (e.g., Refs. [61,62]) show that people tends to compare the current weather conditions with those from recent past experienced by them directly or indirectly. In this context, the use of standard climate period would assure scientific accuracy, but it would not fully answer to the users' needs and/or expectations (e.g., significant differences between the climatic information and the current weather conditions). Therefore, the use of a shorter but more recent time period may be better fitted for deriving the climate information for a tourism-oriented product. Going further, the presentation form should include ‘translation’ of the information, that is to use intuitive, easy to understand graphics possibly associated with short text explanations, while preserving the scientific quality. The delivery method should insure a single-point access, this being in fact one of the major advantages of the service; disparate information potentially useful for tourism may be found in different locations/access points, but the service would insure that all the relevant information is easily accessible through a single point/source. Regarding the free/paid access to the climate service content, available studies (e.g., Refs. [12,31]) suggest that only a limited pool of users would pay for this type of information, but this issue may be dealt with by adding more specific products, by extending the users pool (e.g., information customized not only for tourists but also for tourists investors and decision-makers etc.).

Applying these conditions to thermal stress seen as a potential tourism-oriented climate product, the population interest for this information [12] assures that it may be considered a good choice to be included in a climate service oriented towards tourists. To insure its uptake, the thermal-stress product should be associated with a high scientific quality as a fundamental requirement to build the user trust. Furthermore, it should bring a significant added value to the tourists (e.g., to be valid at destination scale, for the period/timescale of interest for tourist etc.). In assuring these characteristics for the thermal stress product but also for any tourism-oriented climate product there are however limitations emerging from the data characteristics – for input data but also for the data needed to assess the product performance.

The input data refers to the climate information needed to compute the product; as measurements of weather parameters are clearly not available in all potential destinations, alternative data sources should be used, like for example reanalysis data, gridded data derived from observations or climate models outputs. The consistency between all the input data (i.e., all parameters of interest) should be assured, this leading usually to the use of a single dataset containing all the necessary parameters. The spatial scale is another data-related issue to be considered. While for most climate-related applications the spatial scale of 10–25 km is relevant, the requirement to produce information at destination level (i.e., specifically for that locality) leads to the necessity of using data at finer spatial scales – for example at 1 km for small villages or mountain areas to 3–5 km in the case of large urban areas. However, little climate data is currently available at such spatial scales, thus alternative technical options should be applied (e.g., downscaling the available data at the desired scale) or a compromise between the requirements and the data characteristics should be found.

On the other hand, data is needed also for assessing the performance of the product. The specific objectives of the assessment may be derived from the need to demonstrate and communicate the benefits [31] and they are of more general relevance. In reaching any of these objectives (OBJ), several potential approaches (PA) may be employed and some of these are exemplified in the following, with a focus on the biometeorological product based on thermal stress.

- OBJ: asses the relevance for tourism actors. PA: The **assessment of user uptake** would be the first way of estimating how relevant is the product for the users, however this analysis would refer mostly to the service as a whole than to individual products. An alternative solution would be the **analysis of users feedback** referring directly to the product evaluated. This would require a sufficiently long time of service availability, associated with a sufficient number of specific feedbacks. The data required by this approach is not yet available either for WeCENT prototype service or for other climate service targeting individual tourists. Therefore, this approach is not feasible at the moment to evaluate the thermal stress-related product.
- OBJ: highlight the relevance for tourism sector. PA: **comparison against touristic data in order to assess the relationship between climate-related information described by the product and tourism flux (**e.g., if it influences/it correlates with sectoral data). In the particular case of thermal stress, this approach could allow to use the product in a forecast manner to inform the decision makers (e.g., authorities, managers, investors) and help them to better plan their activities; it would also contribute to answer, at least partially, to users' needs as identified by Ref. [26] and to the interest of hotel managers for how climatic conditions at the departure point may influence the tourist arrival. However, as products related to thermal stress are not currently used for tourism purposes -at least not in a ‘direct’ manner, which would allow to quantify the relationship, such a comparison, at this time, would not lead to relevant findings regarding their applicability for tourism and therefore it would not be applied in this study. This approach may nevertheless be relevant, for example, in case of launching such products on the market (e.g., within an application with a large pool of users); doing this type of analysis after a certain time may confirm or not the relevance of such products among the users of the application, thus providing support information on further development of the application.
- OBJ: investigate the relevance of data for other sectors. PA: **relating the indices to biological effects observed in (selected) touristic destinations**, **in order to establish the performance of the indices in describing the observed response in tourists' health.** Such an approach could contribute to the risk management situations at the destination level, by providing supplementary information for the local authorities in planning the emergency situation responses in relation to tourism flux (e.g., availability of proper medical services, tourists information on the subject). However, the necessary data is not easily available, especially taking into account that additional (personal) factors involved in the observations (e.g., pre-existing health issues, personal conditions etc.) should be available and considered in the analysis. Given these limitations, this approach is not feasible, at the moment, to be applied for evaluation the thermal stress product.
- OBJ: highlight the product (technical) quality. PA: **comparison with existing similar data**, where available, **in order to evaluate the advantages and/or limitations of products developed compared to a ‘reference’ dataset.** A dataset on UTCI computed at European level is freely available from CDS [63]. This dataset is derived from global climate reanalysis ERA5 and it has a spatial resolution of nearly 25 km, compared to about 11 km in UERRA. The coarser resolution of ERA5 dataset is expected to bring low added-value from a tourist’ perspective, which requires information at finer spatial scale (i.e., for a certain destination). Furthermore, the method for computing UTCI is different from the one employed in the present study. Consequently, the comparison between the two datasets may provide useful information for example on the impact of horizontal resolution and on the computation methods of UTCI values, but it would not allow assessing the contribution of each of the investigated factors (i.e., horizontal resolution, UTCI computing method) in the results of the comparison. Therefore, this approach is not applied in this study for the evaluation of the thermal stress product.
- OBJ: assess the impact of technical (data) constraints. PA: **comparison of main parameters** employed in computing the product and provided by different datasets **in order to assess the impact of input data on the accuracy and relevance of the product**, which in turn may influence the user uptake of such a product. The advantage, compared to the previous approach, would be that the analysis focuses on only one factor – the data employed. Another advantage relies on the availability of input data, making it feasible also for the tourism-oriented climate product based on thermal stress.

This latter approach may be applied practically to any of the weather parameters used for computing the climate information in the product. In the case of the thermal stress-related product, air temperature is one of the main influencing physical parameters on outdoor thermal comfort [64]. Wang et al. (2022) [65], in a study on thermal comfort experienced by virtual tourists, found that thermal comfort was highly correlated with environmental temperature and ‘In the state of exercise (slow walking, fast walking), the environmental temperature affected tourists’ physiological indicators'.

Hence, for the performance analysis of the thermal-stress related climate product aiming to assess the impact of data constraints, the last approach is applied and presented in the following. Its implementation targets the assessment of the effect produced by the use of various temperature-related parameters in the computation of thermal stress. This approach is also close to the objective of the study, by providing insight on possible future developments/enhancing of the quality of the product.

## 4 Evaluation of the climate product based on UTCI – impact of input temperature data

The performance analysis is based on the comparison of the thermal stress product included in WeCENT prototype service and considered as reference, against thermal stress computed with two different temperature-related indices namely daily maximum and daily mean temperature, the latter being considered as test products. The analysis addresses only to the impact of temperature-related input data, thus all the other parameters are the same in all products (reference and test ones). The results are analyzed with a particular focus on the number of days in the ‘pronounced stress’ category, highlighting the change in this index for each test product compared to the reference product.

### 4.1 Data and methods

The UTCI index was implemented by making use of.

• Information from COST 730 project [66] which provided the framework for the development of the index. UTCI is calculated as a 6th order polynomial regression function of T2m, W10 m, e (vapour pressure) and the difference (Tmrt-T2m).
• information from BioKlima2.6 software [67] - in particular information on how to compute Tmrt; in the Tmrt formula, there is a quantity representing 'Solar radiation absorbed by nude man' for which BioKlima proposes 3 options to compute it, depending on what type of input data is available; the procedure implemented for this study uses the version based on cloud cover (SolAlt model).

The procedure also includes a part which allocates the UTCI value in one of the stress categories (Table 1, columns 1 and 2), such that the result may be expressed either as a value or as a stress class/category. In the view of using UTCI as basis for a tourist-oriented climate product, it is expressed using three categories of thermal stress intensity, showed in the left-side column of Table 1.

In the reference product (i.e., included in the WeCENT service prototype) UTCI index is derived from the European regional climatic reanalysis dataset UERRA [68]. The dataset is freely available from Copernicus Climate Data Store (CDS) [17] and it has a spatial resolution of about 11 km. The reference product is derived for the period 2000–2018 from daily data and it is aggregated at monthly scale, the final information being presented in terms of monthly mean number of days characterized by the three thermal stress categories presented in the third column of Table 1. The parameters used for computing UTCI are air temperature at 2 m (T2m), relative humidity at 2 m (RH), wind speed at 10 m (w10 m), surface temperature (Tskin) and they represent the instantaneous values at 12:00 UTC. This choice is based on considering that 12:00 UTC is relevant for daily atmospheric conditions as well as for tourism purposes. Also, Di Napoli et al. (2019) [46] showed that heat stress expressed through UTCI has a diurnal pattern, with higher values at 12:00 UTC or 15:00 UTC over Europe. However, the choice of using this data specifically (i.e., instantaneous data at 12:00 UTC) implies limitations, as the maximum temperature is usually reached later during the day which in turn may affect the daily maximum value of thermal stress.

For the test products, the best candidate dataset identified is ‘E-OBS daily gridded meteorological data for Europe from 1950 to present derived from in-situ observations’ (further denoted as E-OBS) available from Climate Data Store (CDS) [69]. This datasets has the same spatial resolution as UERRA (about. 11 km).

The analysis is confined at a limited number of locations – namely for the 10 urban areas included in WeCENT (Table 2) and only for summer months. This latter choice is based on the fact that thermal stress may be amplified by the urban environment through the Urban Heat Island (UHI) effect and thus assessing the effect of input data on UTCI computation may be of most interest in such areas, especially in the view of a potential commercial exploitation of the product/service. Also, the association between thermal stress and health effects is stronger for hot conditions, as pronounced thermal stress in hot conditions has been associated with an increase mortality (e.g., Refs. [46,70]).

**Table 2:** *Selected urban locations employed in the analysis*.

Locality	Latitude	Longitude	Altitude [m]	Resident population (at Jan 1, 2023) according to national statistics (http://dati.istat.it/; www.insse.ro)	Köppen – Geiger Climate classification
Bolzano	46.49	11.33	262	533 267	Cfb (Oceanic Climate)
Napoli	40.86	14.25	17	2 969 571	Csa (Hot Summer Mediterranean climate)
Firenze	43.78	11.24	50	984 991	Csa (Hot Summer Mediterranean climate)
Milano	45.46	9.18	120	3 219 391	Cfa (Humid Subtropical Climate)
Torino	45.08	7.67	239	2 198 237	Cfa (Humid Subtropical Climate)
Oradea	47.06	21.93	142	217 521	Dfb (Warm Humid Continental Climate)
Alba Iulia	46.06	23.58	330	74 447	Dfb (Warm Humid Continental Climate)
Iasi	47.17	27.57	60	394 546	Dfb (Warm Humid Continental Climate)
Targu Jiu	45.05	23.28	250	91 847	Dfb (Warm Humid Continental Climate)
Braila	45.28	27.97	25	189 486	Dfa (Hot Humid Continental Climate)

The performance analysis thus focuses on the comparison of the impact on the thermal stress intensity of three parameters related to air temperature at 2 m (T2m): (1) instantaneous values of T2m at 12:00 UTC from UERRA; (2) daily maximum (Tmax) air temperature from E-OBS and (3) daily mean temperature (Tmean) from E-OBS. The impact of input air temperature on the thermal stress intensity is expressed through the change in the monthly mean number of days with ‘pronounced thermal stress’. The comparison is performed for the period 2011–2018, when both datasets (UERRA and E-OBS) are available.

### 4.2 Results

In order to estimate the impact of different temperature-related input data on the thermal stress intensity UTCI is computed with E-OBS daily mean and daily maximum temperature data, while the other parameters are still provided by UERRA and thus they represent instantaneous values at 12:00 UTC.

Taken into account the different source, meaning and representativity of the meteorological parameters included in UTCI in this way, this should be considered just a sensitivity exercise and not a real possibility of computing the thermal stress. Nevertheless, the exercise allows to estimate the magnitude of the change in the final product and may provide future insight on the methodology for computing this information.

The comparison between the reference product (i.e., UTCI computed from UERRA) and the test ones (i.e., UTCI computed with Tmax and Tmean from E-OBS and all other parameters from UERRA) focuses on the changes of the monthly mean number of days of ‘Pronounced hot’ stress class, as defined in Table 1.

**Fig. 1:**
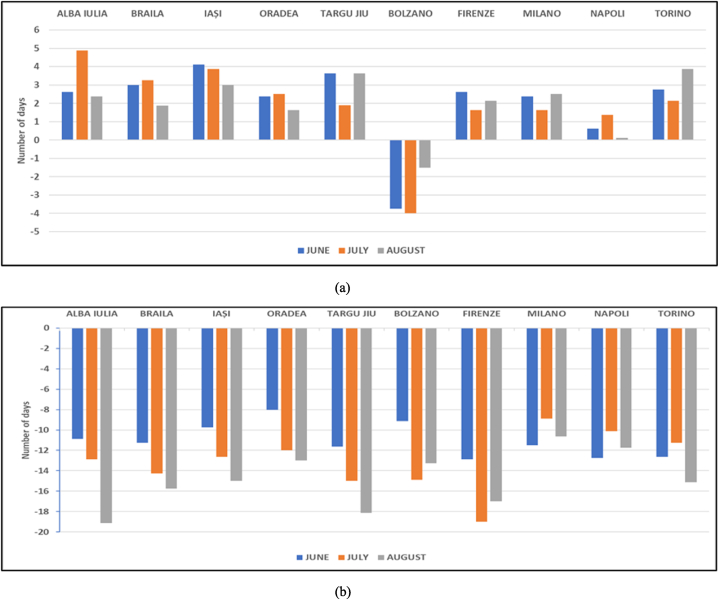
Differences in the monthly mean number of days in the ‘Pronounced hot stress’, for period JJA 2011–2018, due to the use of (a) E-OBS daily maximum temperature and (b) E-OBS daily mean temperature compared to the use of instantaneous air temperature at 2 m at 12:UTC from UERRA.

The results are shown in Fig. 1 for each month and each urban destination. It may be seen that using daily maximum temperature in computing UTCI (Fig. 1a) leads to the increase of the monthly mean number of days with intense thermal stress for all locations except Bolzano. The most noticeable increase is in general in June, with around four more days in this class for Iași and Târgu Jiu and up to 3 more days for Brăila and Torino, while the smallest change is observed for Napoli. On the contrary, the use of daily mean temperature (Fig. 1b) leads to a strong decrease in the monthly mean number of days with pronounced hot stress (e.g., about 19 days less in August at Alba Iulia compared to the reference product); practically, the thermal stress ‘becomes’ less intense, describing more favorable climate conditions than any of the other two cases.

**Fig. 2:**
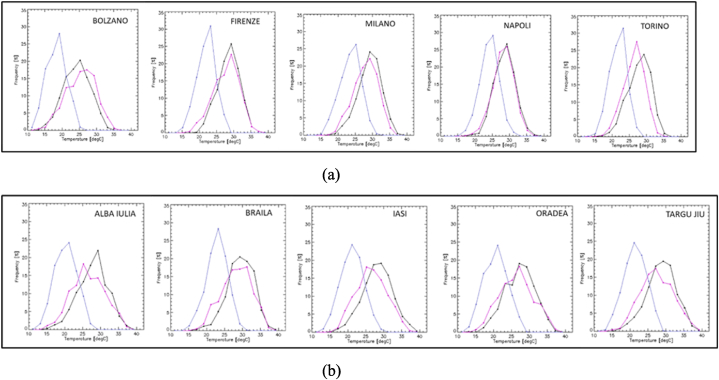
Frequency of distribution, for June–August 2011–2018, of daily maximum temperature from E-OBS (black line), daily mean temperature from E-OBS (blue line) and instantaneous values of air temperature at 2 m at 12:00UTC from UERRA (pink line), for (left to right): (a) Bolzano, Firenze, Napoli, Firenze, Milano and Torino; (b) Alba Iulia, Braila, Iasi, Oradea and Targu Jiu. (For interpretation of the references to colour in this figure legend, the reader is referred to the Web version of this article.)

The signs of changes in the test products compared to the reference one are to be expected if looking at the frequency distribution of the respective input temperatures (Fig. 2 a,b). It may be seen that in general in UERRA dataset the values are more frequent around the interval 25–27 °C, compared to daily maximum values in E-OBS, for which the classes with higher temperatures are more populated. On the other hand, daily mean temperatures in E-OBS are noticeably lower than any of the other two temperature parameters, with most populated classes around 17–23 °C.

## 5 Discussions

The use of any of the three temperature-related data types employed so far may be justified, as they are all used in various applications. The sensitivity exercise may be extended with other types of temperature-related data – as for example using measured temperature or mean/maximum temperature over a certain time interval (e.g., daylight hours). These choices would also be associated with limitations like for example the impact of UHI effect [71], the details in the spatial pattern of temperature in the cities due to land-use type [72], assumptions related to the definition of the time interval. However, the impact of using a certain type of input data should be considered from the tourists' perspective. In this line, the differences in the final thermal stress information discussed before are quite important given the potential negative effects on health of increased thermal stress and they should be considered in the quality analysis of the product to be included in a climate service. In the particular case of the computational choice employed for the reference product used in this study and included in the WeCENT project, these findings have been taken into account by introducing a short ‘warning’ phrase in the final version of ‘Thermal stress’ product available for urban, rural and seaside touristic destinations [28]: ‘It should be noted that the thermal stress may be more intense at certain moments during the day than it is described here.’

As a prerequisite for a good user uptake, the product provided through a tourist-oriented climate service should be as accurate as possible in the limits imposed by the data characteristics. Among the solutions to improve the accuracy of the product based on UTCI, in the framework presented in the study, might be considered the following.

- to use daily maximum/minimum values from UERRA forecast products. UERRA reanalysis are available only at 00:00, 06:00, 12:00 at 18:00 UTC; simple mean of these, as proxy for the daily mean, leads to lower values than those at 12:00 UTC, as confirmed during the study. However, forecast products are also available and these might provide more accurate input for temperature.
- to use national meteorological database, which contains all the required parameters, these fulfilling the conditions described before (e.g., consistency, temporal frequency etc.) and contributing to a higher accuracy and relevance of the product.

The data characteristics are not the only limiting factor of the UTCI-based product accuracy and/or relevance. Other sources of uncertainty, leading to possible downfalls in the product ability to describe the subjectively sensed thermal stress, regard the representativity of UTCI assessment scale (i.e. not taking into account the people acclimatization) [73], the computation of some physical parameters used in UTCI (e.g. mean radiant temperature, radiation fluxes) [74] or limitations related to special meteorological conditions (i.e. low temperatures and high wind situations) [75]. As these limitations cannot be fully eliminated, they should be properly communicated to the product users in order to strengthen their confidence in the quality and relevance of the scientific information provided.

## 6 Conclusions

The study addresses the characteristics of a climate service targeting tourists and discusses the evaluation of its products, including the analysis of end-users requirements, definition of specific objectives of the evaluation, identification of the ways to reach them (i.e., approaches), identification of data needed to implement the selected method/approach. Furthermore, an assessment of the input data on the accuracy and relevance of a product is presented for the particular case of thermal stress seen as a tourism-oriented product, expressed through UTCI. The analysis is based on the use of three types of temperature-related information – instantaneous temperature at 12:00 UTC, daily maximum temperature and daily mean temperature - applied for 10 urban destinations located in Italy and Romania. The results show that the monthly mean number of days with pronounced thermal stress may increase with up to 4 days in some of the selected 10 urban locations when using daily maximum temperature for the computation of thermal stress, while the use of daily mean temperature may lead to a strong underestimation of the thermal stress intensity.

The study contributes to the research literature targeting the evaluation of climate services on two directions: firstly, by discussing a less-common type of such service – namely a service targeting a segment of the general public rather than authorities or decision factors; secondly, by focusing on the evaluation of the content elements of the service (i.e., products) as an alternative to the evaluation of the whole service. Furthermore, the methodology may be customized for any climate product targeting tourists.

The results of the performance analysis of thermal stress seen as a tourism-oriented climate product in relation to the input data highlight a strong impact on the final output, which should be considered from the perspective of its relevance for end-users. In particular, the results suggest that the use of daily maximum/minimum temperature for warm/cold season would be the best choice for deriving thermal stress for tourism purposes. Although this method may lead to apparently less favorable climate conditions, it would answer better to tourists interests (e.g., information on the probability to experience extreme weather/climate conditions) and it would contribute to a safer touristic experience.

The findings of the study are associated with limitations which may come from several directions. The most evident limitations are related to the reduced number of locations employed in the analysis and to the use of a single alternative dataset to investigate the impact of temperature-related data on characterization of thermal stress intensity. Additionally, choices related to the UTCI computation (e.g., use of polynomial regression vs LUT approach; computation at daily scale instead of hourly frequency) may also limit the accuracy of some results. The relevancy of UTCI assessment scale is another direction still open for discussions in literature and therefore a possible source of limitation for the outcomes of this study. Recent literature shows that the subjective sensations related to perceived thermal stress may vary for different populations [76] or may be influenced by the departure and arrival destination climate [77] and thus by the individual resilience to climate/weather conditions or they may be influenced even by the specific location/place of touristic activity [78]. The choice used in this study on the aggregation scale in a smaller number of comfort categories of the UTCI associated assessment may also influence the relevancy of the results. Also, the use of UTCI instead of another tourism climate index may contribute to the limitations associated with this study.

It should still be noted that there is no ‘receipt‘ for building a tourism-oriented climate product based on thermal stress. While several indices are available for deriving such information, even adapted for specific regions (e.g., Mediterranean Outdoor Comfort Index (MOCI) [79]) the other characteristics of such a product are not fully described so far and therefore they are subject to ‘trial and error’ approach. Furthermore, in designing a successful tourism-oriented climate service, especially in the case of market exploitation, a trade-off should be found between the efforts for developing the products included in the service (e.g., amount and type of input data, its accuracy, number and types of indices, temporal frequency etc.) and the ‘gain’ obtained through its outcomes (e.g., intensity of user uptake, economic gain).

Future research will address some of the limitations identified previously in order to provide a more comprehensive view of the optimal choice of input data able to assure a high accuracy and relevance of the product. Also, additional approaches for the performance analysis will be employed (e.g., comparing with existing similar data; user uptake) allowing to gather more information for future development of tourist-oriented climate products.

## Funding

This project received funding from the European Union's 10.13039/501100007601Horizon 2020 research and innovation program under the Marie Sklodowska–Curie Grant agreement no. 887544.

## CRediT authorship contribution statement

**Liliana Velea:** Writing – original draft, Formal analysis, Conceptualization. **Zenaida Chitu:** Writing – review & editing, Resources, Investigation. **Roxana Bojariu:** Writing – review & editing, Validation, Investigation.

## Declaration of competing interest

The authors declare that they have no known competing financial interests or personal relationships that could have appeared to influence the work reported in this paper.
